# The Role of Kv1.2 Channel in Electrotaxis Cell Migration

**DOI:** 10.1002/jcp.25259

**Published:** 2015-12-10

**Authors:** Gaofeng Zhang, Mathew Edmundson, Vsevolod Telezhkin, Yu Gu, Xiaoqing Wei, Paul J. Kemp, Bing Song

**Affiliations:** ^1^ Department of DermatologyNo. 1 Hospital of China Medical UniversityShenyangChina; ^2^ School of DentistryCollege of Biomedical and Life SciencesCardiff UniversityCardiffUnited Kingdom; ^3^ School of BiosciencesCollege of Biomedical and Life SciencesCardiff UniversityCardiffUnited Kingdom

## Abstract

Voltage‐gated potassium Kv1.2 channels play pivotal role in maintaining of resting membrane potential and, consequently, regulation of cellular excitability of neurons. Endogenously generated electric field (EF) have been proven as an important regulator for cell migration and tissue repair. The mechanisms of ion channel involvement in EF‐induced cell responses are extensively studied but largely are poorly understood. In this study we generated three COS‐7 clones with different expression levels of Kv1.2 channel, and confirmed their functional variations with patch clamp analysis. Time‐lapse imaging analysis showed that EF‐induced cell migration response was Kv1.2 channel expression level depended. Inhibition of Kv1.2 channels with charybdotoxin (ChTX) constrained the sensitivity of COS‐7 cells to EF stimulation more than their motility. Immunocytochemistry and pull‐down analyses demonstrated association of Kv1.2 channels with actin‐binding protein cortactin and its re‐localization to the cathode‐facing membrane at EF stimulation, which confirms the mechanism of EF‐induced directional migration. This study displays that Kv1.2 channels represent an important physiological link in EF‐induced cell migration. The described mechanism suggests a potential application of EF which may improve therapeutic performance in curing injuries of neuronal and/or cardiac tissue repair, post operational therapy, and various degenerative syndromes. J. Cell. Physiol. 231: 1375–1384, 2016. © 2015 The Authors. *Journal of Cellular Physiology* Published by Wiley Periodicals, Inc.

Endogenous electric field (EF) is crucial for the development of nervous system, stem cell differentiation, and wound healing (Zhao et al., 2006; Reid et al., 2009; Cao et al., 2013). One of the most essential biological effects of the endogenously generated EF is induction of directional cell migration. Many cell types were observed to be able to respond to the applied EFs of physiological strength with directed cell migration (McCaig et al., 2005). Cell migration is vital to a wide range of biomedical events such as wound healing (Martin, 1997; Chi and Trinkaus‐Randall, 2013), inflammatory response (Kolaczkowska and Kubes, 2013), and cell‐based therapies (Bulte et al., 1999; Moraes et al., 2012). Whilst migratory cues such as chemoattractants and mechanical stress have been widely studied, the mechanisms underlying how cells sense and move in response to EFs (“electrotaxis”) still remain poorly understood. As different cells show different responses to the similar exogenously applied EF stimuli—moving either towards the cathode or the anode (Mycielska and Djamgoz, 2004), the transmembrane ion currents were suggested to be associated with such processes (Nuccitelli, 1988). It has been observed that intracellular Ca^2+^ concentration increase resulted in the activation of contractility of *Dictyostelium* cells when treated with EF (Shanley et al., 2006). In parallel with additional experiments which proved that Ca^2+^ blocker inhibits electrotactic response of the cells, the alternative Ca^2+^ independent mechanism has also been reported in the regulation of electrotaxis as well (Huang et al., 2009). For example, it was demonstrated that Na^2+^ channels were required in the prostate cancer metastasis via an electrotactic effect (Djamgoz et al., 2001). However, little is known about the role of other potential ion channels in the regulation of electrotaxis.

Voltage‐gated potassium (K^+^) channels contribute to the regulation of membrane potential and, consequently, to cell excitability (Edwards and Weston, 1995). K^+^ channels are known to be involved in non‐electrotaxis driven cell movement (Schwab et al., 2008), for example, Ca^2+^‐activated K^+^ channels (K_Ca_3.1) are essential for dendritic cell migration (Shao et al., 2011). Kv1.2 channel or “shaker” is an attractive candidate as an EF sensor, since it is involved in the regulation of numerous voltage‐sensing pathways including regulation of resting membrane potential, propagation of excitation in the nerves (Shen et al., 2004), and in controlling of the heart rate (Chen et al., 2010). Like many other types of voltage‐gated K^+^ channels, Kv1.2 channel has a trans‐membrane voltage sensing domain with four arginine residues so that shifts of membrane potential result in sequential changes of the channel's conformation which is followed by pore opening (Long et al., 2005). Furthermore, Kv1.2 channels are able to bind cytoskeleton components arranging protein partnerships and mediate cell motility (Uruno et al., 2001).

Put together, we hypothesize that voltage‐gated Kv1.2 channels may play an important role in the regulation of electrotaxis, and investigated the mechanism of Kv1.2 in this event. Three clones of COS‐7 cells overexpressing Kv1.2 genes were generated to test the function of Kv1.2 channel in electrotaxis. Loss of function analysis were performed using Kv1.2 channel inhibitor, charybdotoxin (ChTX). In this study we also analyzed the interaction of Kv1.2 channels with the downstream signalling molecule‐actin binding protein cortactin, and possible mechanisms controlling directed cell migration. We propose that Kv1.2 channels play EF‐sensor and transduce signals via cortactin, which sequentially promotes the electrotactic response of the cells.

## Materials and Methods

### COS‐7 cell lines culture and generation of stable Kv1.2‐expressing clones

COS‐7 cells were maintained by culturing the cells in DMEM/F12 1:1 medium containing l‐glutamine 15 mM HEPES (Gibco, Paisley, UK) with added 10% FBS and (100 ug/ml) penicillin, (100 U/ml) streptomycin (Gibco, Paisley, UK). COS‐7 cells were cultured on Sarstedt plastic ware at 37°C, 5% CO2 and passaged every 2–4 days at 80% confluence. The Kv1.2 gene was cloned from purified mouse genomic DNA into the expression vector pcDNA3.1. COS‐7 cells were transfected with the vector containing the Kv1.2 gene using a FuGene transfection kit (Promega, Southampton, United Kingdom). To generate and maintain a stable transfection 1% geneticin (Life Technologies, Paisley, UK) was added to the normal culture medium to select for cells successfully incorporated pcDNA3.1.

### EF application, time‐lapse imaging, and data processing

EFs were applied in electrotaxis chambers similar to the previously described (Zhao et al., 1996; Song et al., 2007). Briefly fibronectin (Sigma, Poole, UK) was applied to a 4 cm^2^ area marked on a petri dish (0.5 μg/cm^2^) to create a matrix for cell attachment. COS‐7 cells were seeded onto the fibronectin at approximate density of 5 × 10^4^ cells/cm^2^ and incubated for 20–30 min at 37°C, 5% CO_2_ to allow the cells to bind to the fibronectin. A glass coverslip was then used to cover the cells and seal the electrotactic chamber. Agarose‐salt bridges (2% agarose gelled in Steinberg solution) were used to connect beakers of Steinberg solution to the culture medium reservoirs at either side of the electrotactic chamber. Silver chloride electrodes were placed into the beakers containing Steinberg solution and connected to the EF power supply. EF was applied (ranging from 0 to 300 mV/mm) and time‐lapse imaging performed using a DeltaVision imaging system (Imsol Ltd, Preston, UK). Real‐time image were recorded at 5 min interval for 4 h. The collected data were analyzed using ImageJ software with the average speed, distance and direction of movement, and minimum 200 cells were measured per experiment. The average direction or “directedness” of the cells was calculated and expressed as a Cosθ value, with a Cosθ of 1 indicating all the cells were moving towards the anode on the right of the screen and Cosθ of −1 being to the cathode, on the left. Zero (0) value indicates random movement, that is, no or chaotic movement towards either anode or cathode.

### Western blotting and immunoprecipitation

Post EF stimulation, COS‐7 cells were immediately trypsinized and extracted from the electrotactic chamber, washed in ice‐cold PBS and incubated with lysis solution on ice for 10 min. Cell debris was removed by centrifugation at 16,000*g* for 5 min. Protein concentration was determined using a BCA assay kit (Thermo Scientific, Loughborough, UK). Protein samples were mixed with loading buffer (Novex, Paisley, UK) and denatured at 70°C for 5 min. The proteins were separated with 4–2% Bis‐Tris polyacrylamide gels (Life Technologies, Paisley, UK), and transferred onto nitrocellulose membranes (Novex, Paisley, UK). After washing the membrane in 1xTBST and blocking with 5% non‐fat milk the appropriate primary antibody was applied at the recommended concentration and incubated overnight at 4°C. The membranes were washed and re‐blocked before appropriate secondary antibody (conjugated to horseradish peroxidise) was incubated at room temperature for 1 h on a shaking platform. The blots were then processed with chemiluminescent substrate (Geneflow, Staffordshire, UK) and exposed to X‐ray films.

Immunoprecipitation pull‐down analysis was carried out using protein A/G agarose beads (Santa Cruz, Middlesex, UK). COS‐7 equal amount of proteins were loaded onto the beads. The pull‐down was performed according to the manufacturer's instructions and the proteins obtained underwent western blotting as described above.

### qPCR

COS‐7 cells from the control and EF‐stimulated groups were collected with addition of Qiagen cell lysis buffer from the RNAeasy RNA extraction kit (Qiagen, Crawley, UK). The Qiagen kit was used to purify whole cell RNA with the final concentration determined by measuring absorbance at 260 nm on the Nanovue nanodrop system (GE Healthcare Ltd, Hertfordshire, UK). Total cell mRNA was reverse transcribed using master mix supplied by Primer Design (Southampton, UK) and a reverse transcriptase from Promega (Southampton, UK). Hundred nanogram equivalent of cDNA from this reaction was added to a 96‐well qPCR plate (Primer Design, Southampton, UK), followed by the recommended amounts of qPCR reaction components (Primer Design, Southampton, UK). The qPCR was carried out on QuanStudio 6 PCR machine (Life technologies, Paisley, UK).

### Immunostaining and co‐localization analysis

COS‐7 cells were treated as previously described (Chen et al., 2002). Briefly, COS‐7 cells were fixed with 4.0% formaldehyde in PBS at room temperature for 15 min and permeabilized with 0.3% Triton X‐100 in PBS. After blocking with 2% BSA, anti‐Kv1.2 (1:200 dilution; bought from Abcam, Cambridge, UK) and anti‐cortactin (1:200 dilution; Abcam, Cambridge, UK) was applied, and incubated at 4°C overnight. The cells were then washed three times with PBS, and incubated with FITC‐labelled anti‐mouse IgG (1:2000 dilution, Life technologies; Paisley, UK)) or TRITC labelled anti‐rabbit IgG (1:200 dilution, Life technologies; Paisley, UK) at RT (22 ± 0.5°C) for 1 h The slides were mounted in Vectorshield (Vector Laboratories, Peterborough, United Kingdom) mounting medium supplemented with DAPI staining, and registered using Leica confocal microscope imaging system (Leica Microsystems GmbH; Wetzlar, Germany). The co‐localization analysis was performed with ImageJ software.

### Electrophysiological recordings

Macroscopic transmembrane outward currents were recorded using the voltage clamp conventional whole‐cell technique. The bath solution contained (in mM): 135 NaCl, 5 KCl, 1.2 MgCl_2_, 1.25 CaCl_2_, 10 D‐Glucose, 5 N‐2‐hydroxyethylpiperazine‐N′‐2‐ethanesulfonic acid (HEPES); pH was adjusted to 7.4 using 5 M NaOH. The pipette solution contained (in mM): 117 KCl, 10 NaCl, 11 N‐2‐hydroxyethylpiperazine‐N′‐2‐ethanesulfonic acid (HEPES), 2 Na_2_‐ATP, 1 Na‐GTP, 1.2 Na_2_‐Phosphocreatine, 2 MgCl_2_, 1 CaCl_2_, and 11 ethylene‐glycol‐tetra‐acetic acid (EGTA); free [Ca^2+^]_i_ was adjusted to 20 nM; pH was adjusted to 7.2 with KOH. All experiments were done at a controlled room temperature (22 ± 0.5°C).

All electrophysiological studies were performed using an Axopatch 200B amplifier and Digidata 1322A A/D interface (Axon Instruments, Forster City, CA). All recordings were filtered with an 8‐pole Bessel filter at 5 kHz and digitized at 10 kHz. For recording macroscopic currents in conventional whole‐cell voltage‐clamp configuration COS‐7 cells were held at −70 mV and commanded to voltages from −120 to +80 mV in steps of 80 ms duration and increments of ±10 mV. Membrane potential was recorded in normal current‐clamp mode. Series resistance was compensated 60–90%. Pipette resistances were ∼ 7–10 MΩ when filled with the pipette solutions.

### Data analysis

The data were analyzed using Clampfit 9.0, Microsoft Office Excel 2003 and Microcal Origin 6.0 software. Current‐voltage relationships for whole‐cell configuration studies were determined using voltage commands between −120 and +80 mV in 10 mV steps from a holding potential of −70 mV, and steady‐state current densities plotted against command voltage (V_c_). Current densities were calculated by dividing current by capacitance (I/C). ChTX‐sensitive current was obtained by arithmetic subtraction of current in the presence of ChTX from the Control plotted against command potential (mV) and fitted with a Boltzmann equation using an iterative fitting routine:

(1) $$I/C=1/\{1+\exp(V_{0.5}-V_{c})/k\}$$

Conductances (G) were calculated by dividing current by the driving force (V_c_ – E_K_), with E_K_ ∼ −95 mV, plotted against command voltage and fitted with a Boltzmann equation using an iterative fitting routine similarly to the current density:

(2) $$G/G_{\max}=1/\{1+\exp(V_{0.5}-V_{c})/k\}$$

where G_max_ is the extrapolated maximum conductance, V_0.5_ is the voltage (mV) corresponding to half the maximum conductance and k is the slope factor (mV).

Equation (2) was first fitted to the data generated from individual whole‐cell or isolated patch experiments, respectively, the appropriate set of parameters obtained, and then averaged to give the mean values (± SEM). Statistical comparisons of means were performed using one paired or independent *t*‐test; differences were considered significant at *P* < 0.05.

### Chemicals

NaCl, KCL, d‐glucose anhydrites, NaOH, and KOH were purchased from Fisher Scientific UK Ltd (Leicestershire, UK); MgCl_2_, CaCl_2_, ethylene‐glycol‐tetra‐acetic acid (EGTA), and Charybdotoxin (ChTX) were purchased from Sigma–Aldrich Co (Southampton, UK); N‐2‐hydroxyethylpiperazine‐N′‐2‐ethanesulfonic acid (HEPES) was purchased from VWR International (Leicestershire, UK).

## Results

### Characterization of Kv1.2 expression

**Figure 1:**
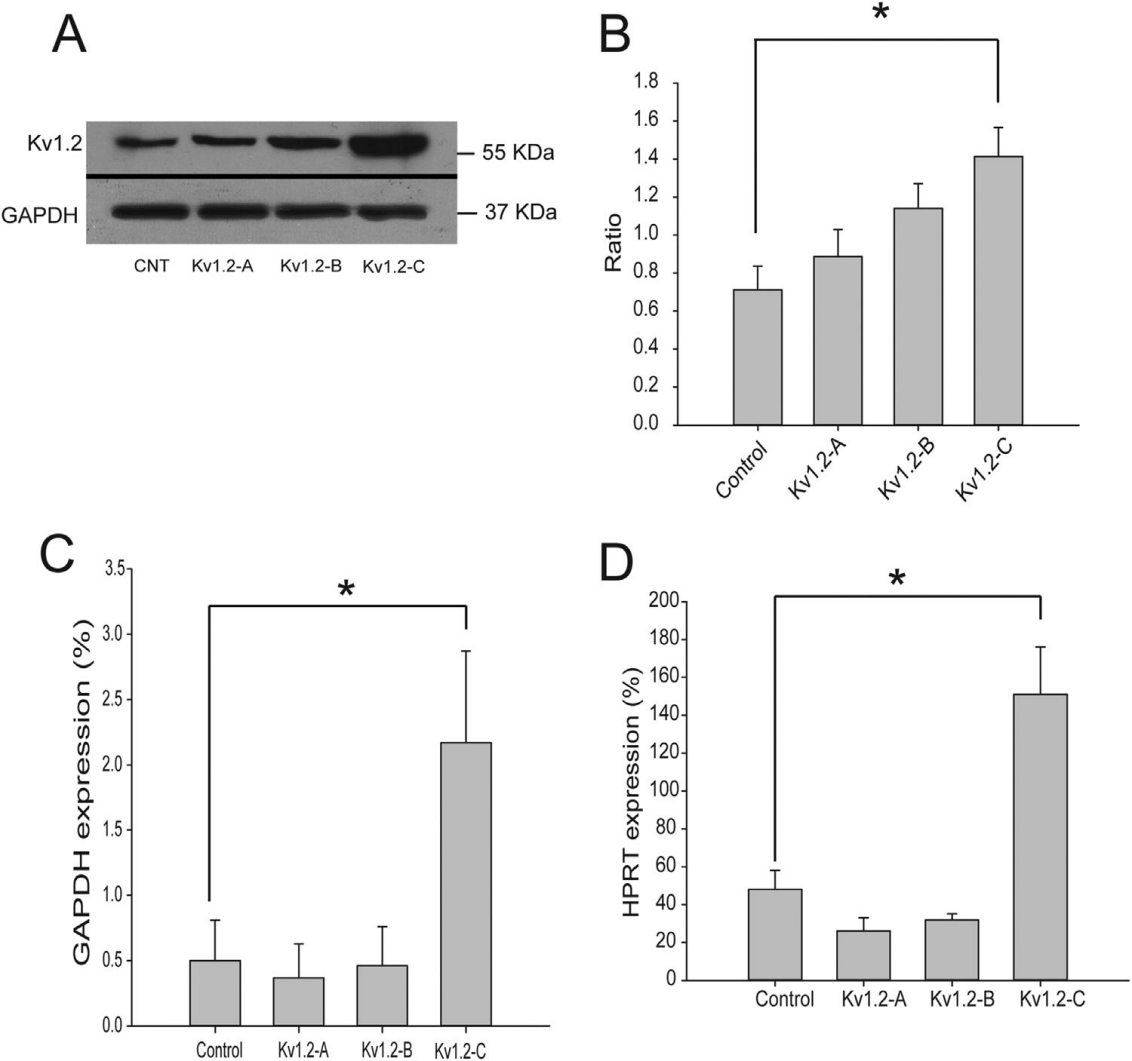
Kv1.2 clone and expression. Mouse K^+^ channel Kv1.2 was cloned into pcDNA3.1 plasmid and three stable Kv1.2 transfected COS‐7 cell lines were generated: Kv1.2‐A, Kv1.2‐B, and Kv1.2‐C. A,B: western blot showed different expression of Kv1.2 in three clones, and expression intensity analysis confirmed that Kv1.2‐C clone had the highest Kv1.2 expression in comparison to WT cells, Kv1.2‐A and Kv1.2‐B clones. C,D: quantitative PCR analysis of Kv1.2 expression in the three Kv1.2 clones confirmed the results in Western blot, comparing to two different house‐keeping genes GAPDH and HPRT, respectively. Results are presented as means ± SE; n ≥ 3. *P < 0.05.

Three COS‐7 cells lines stably expressing mouse Kv1.2 channel (Kv1.2‐A, B, and C) were generated and characterized (Fig. 1). Western blot showed that clone Kv1.2‐C had significantly higher level of Kv1.2 expression than that in wild type (WT) control COS‐7 cells (*P* < 0.05); while Kv1.2‐A and Kv1.2‐B clones had only insignificantly higher levels of Kv1.2 expression compared to WT (Fig. 1A,B). Those results were confirmed by mRNA qPCR analysis (Fig. 1C,D). Compare to both of the reference genes GAPDH and HPRT, only Kv1.2‐C clone displayed significantly higher expression of Kv1.2 than WT which was consistent with the western blot data. Presumably Kv1.2 plasmid was integrated into a position with a high level of constitutive expression in Kv1.2‐C clone.

### Electrophysiological properties of the cells stably expressing Kv1.2 channels

**Figure 2:**
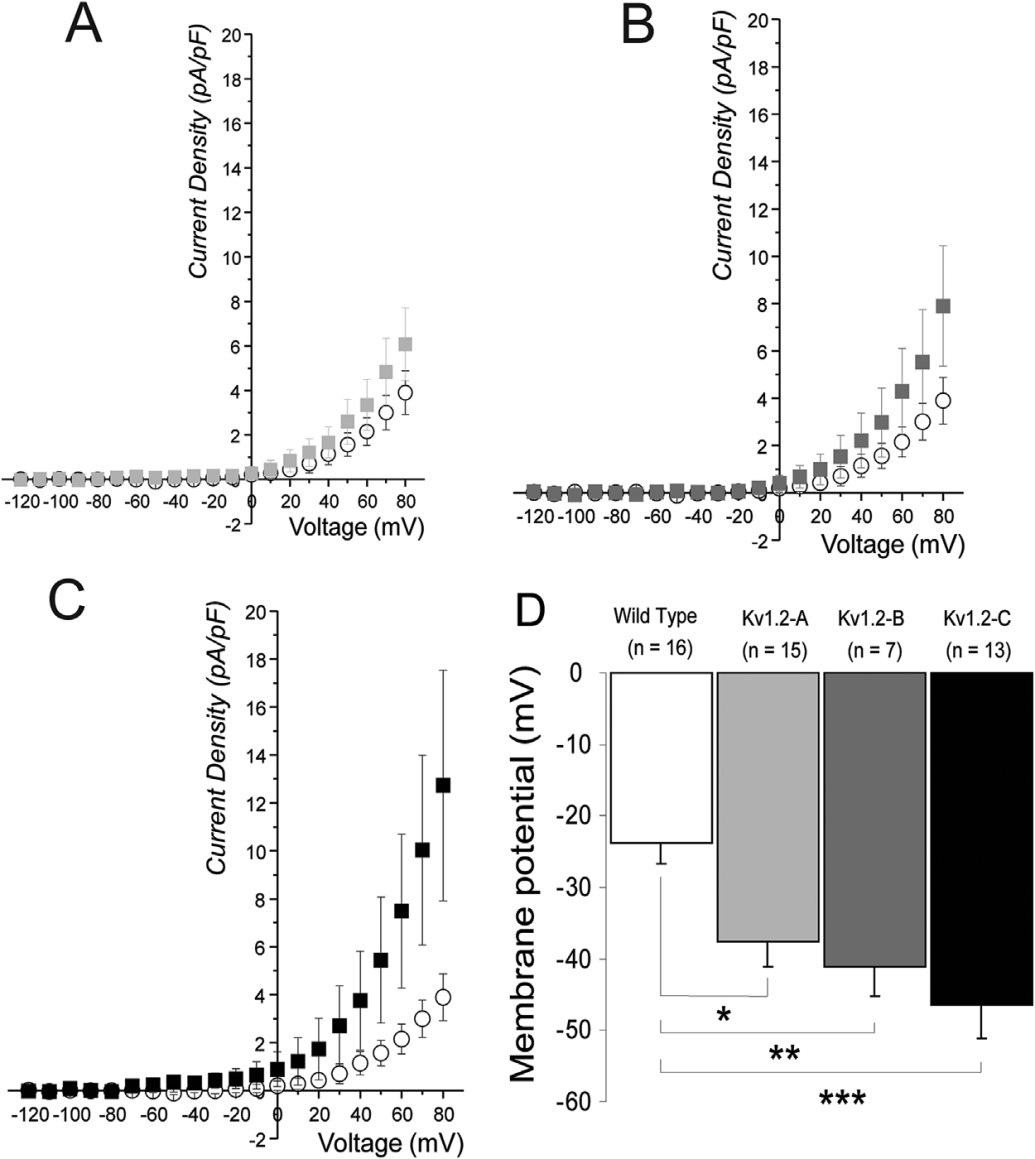
Currents and membrane potential of Kv1.2 Clones recorded in conventional whole‐cell configuration. A–C: mean current densities (pA.pF^−1^) comparison of transmembrane outward currents in COS‐7 WT cells (n = 8), against low (A: Kv1.2‐A, n = 6), medium (B: Kv1.2‐B, n = 5), and high (C: Kv1.2‐C, n = 11) level of Kv1.2 channel expression, respectively. D: diagram of mean membrane potential (mV) of WT COS‐7 cells compared to low, medium and high level of Kv1.2 channel expression (Kv1.2‐A, B, and C). *P < 0.005, ** *P* < 0.003, ***P < 0.0003. Mean ± SE.

Transmembrane current densities (pA.pF^−1^) and membrane potential (mV) were recorded in WT COS‐7 cells and COS‐7 cells with the various levels of Kv1.2 expression: low (Kv1.2‐A), medium (Kv1.2‐B), and high (Kv1.2‐C) by means of voltage‐clamp and current‐clamp, respectively. (Fig. 2 and Table I).

**Table I:** Transmembrane current densities (pA.pF^−1^) and membrane potential (mV) recorded in WT COS‐7 cells and COS‐7 cells with the various levels of Kv1.2 expression

	WT COS‐7	(Kv1.2‐A)	(Kv1.2‐B)	(Kv1.2‐C)
Current densities (pA.pF^−1^)	3.9 ± 1.0 (n = 8)	6.1 ± 1.6 (n = 6)	7.9 ± 2.5 (n = 5)	12.7 ± 4.8 (n = 11)
Membrane potential (mV)	−23.9 ± 2.8 (n = 8)	−37.6 ± 3.4* (n = 6)	−41.1 ± 4.2** (n = 5)	−46.5 ± 4.7*** (n = 11)

The data were considered as significantly different from WT COS‐7: *P < 0.005, **P < 0.003, ***P < 0.0003; Mean ± SE.

Conductance‐voltage curves fitted by Boltzmann function (not illustrated) yielded with a half‐maximal activation voltage (V_0.5_) of 103.1 ± 22.3 mV (n = 8) and 43.2 ± 5.1 mV (n = 11) for COS‐7 WT cells and Kv1.2‐C cells, respectively; the data were considered as significantly different (*P* < 0.01).

### Kv1.2 over‐expression effects on COS‐7 cell migration in EFs

**Figure 3:**
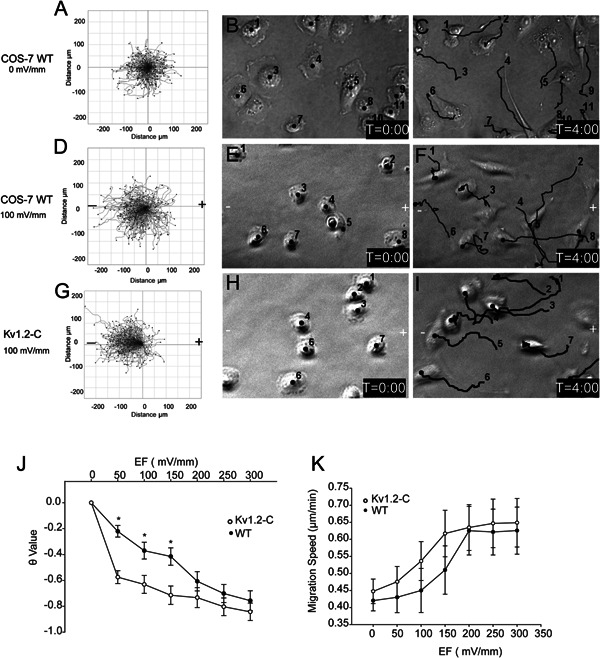
Overexpression of Kv1.2 enhanced electrotaxis of COS‐7 cells. A–I: time lapse imaging was conducted for WT and Kv1.2‐C cells in the absence or presence of 100 mV/mm EF for 4 h, and cell migration behaviors were analyzed with ImageJ software. Cell migration trajectories were accumulated and composed with starting points at the same origin point (0,0) on axes (A,D,G). Middle (B,E,H), and right (C,F,I) columns represent the first and last frame of the real‐time imaging, respectively. The black lines indicate the cell migration trajectories; “–” and “+” indicate the cathode and anode of the electric stimulation, respectively. J–K: Kv1.2‐C clone and WT cells were treated with EF from 0 to 300 mV/mm and their migration directedness (J) and speed (K) were summarized. Results are presented as means ± SE; n ≥ 3, *P < 0.005.

Based on the results described above, Kv1.2‐C clone was selected for the following series of experiments with EF stimulation. Negative migratory control was performed when no chemical or physical cue (e.g., EF stimulation) was applied. Without EF stimulation WT COS‐7 cells moved in random directions; the average directedness of 200 cells without EF stimulation was mathematically determined, giving θ value of 0 for migration; that is, the cells were moving randomly with no overall movement to the left or right (Fig. 3A–C).

When WT COS‐7 cells were exposed to EF of 100 mV/mm in the absence of other physical cues, it resulted with θ value of −0.33, suggesting that WT COS‐7 cells have a slight cathodal bias of migration (Fig. 3D–F). In contrast, Kv1.2‐C cells displayed nearly twice as much migration directedness toward cathode compared to that of WT (with θ value of −0.65), when treated with 100 mV/mm of EF (Fig. 3 G–I).

WT COS‐7 cells and Kv1.2‐C cells were further exposed to EFs at various strengths from 0 to 300 mV/mm (Fig. 3J). Voltage increase yielded significantly greater electrotactic response of each cell type, with nearly 100% of COS‐7 cells moving toward cathode at 300 mV/mm (θ ≈ −1). Kv1.2‐C displayed much more sensitive/faster electrotactic response with much higher cathodal directedness at lower EF stimulation compared to WT COS‐7 cells, and such response almost plateaued at 50 mV/mm. When treated with higher EFs at 200 mV/mm, WT COS‐7 and Kv1.2‐C cells showed identical migration response, suggesting a possible accumulation effect of Kv1.2 channel which saturates the electrotactic response of WT COS‐7 cells eventually when treated with higher EF.

### Kv1.2 inhibitor reduces COS‐7 sensitivity to EF stimulation

**Figure 4:**
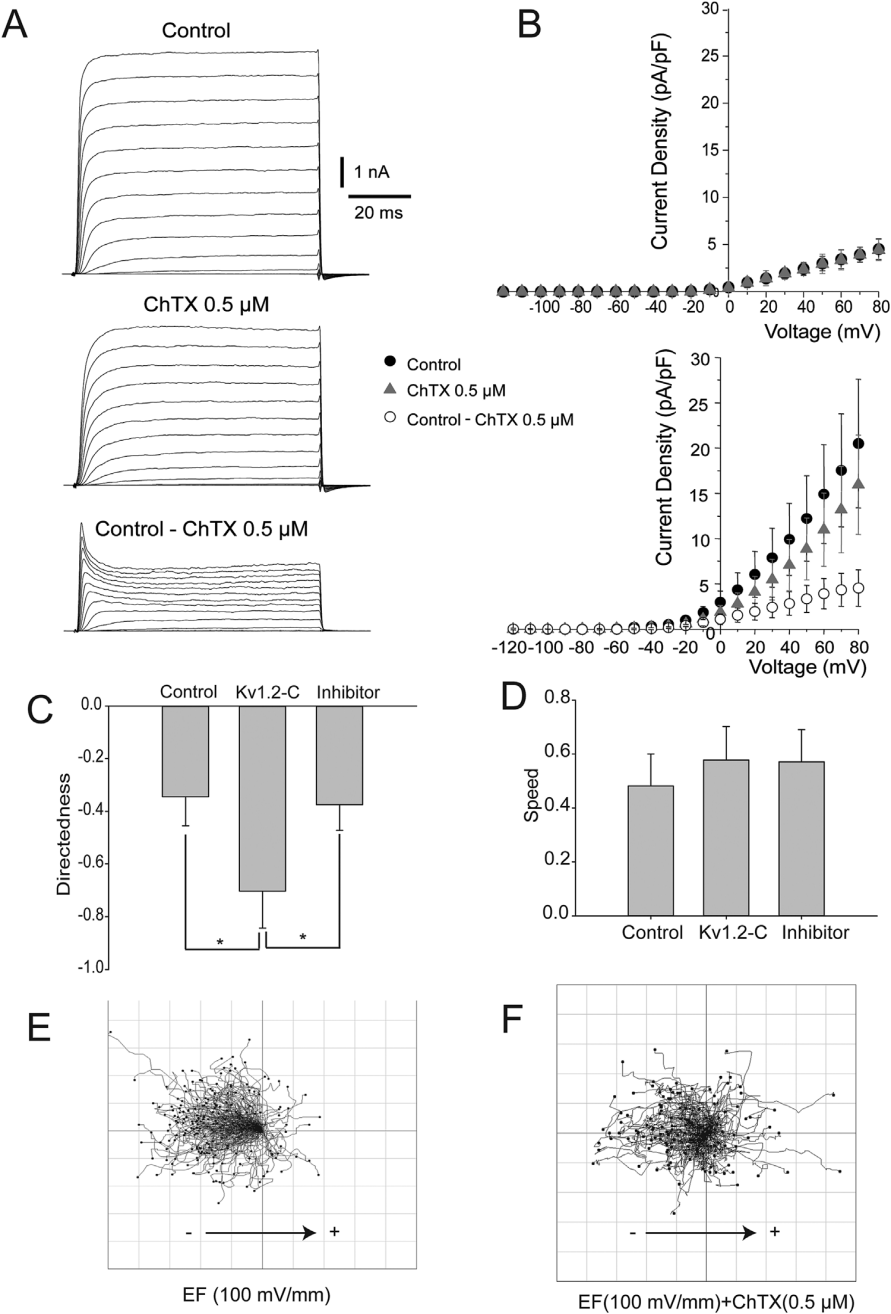
Kv1.2 inhibition diminishes electrotaxis in COS‐7 cells. A: exemplar traces of transmembrane outward currents in Kv1.2‐C clone recorded in conventional whole‐cell configuration generated by 80 ms voltage steps from −120 to +80 mV, in incremental steps of 10 mV from a holding potential of −70 mV in Control (upper part) in the presence of ChTX 0.5 μM (middle part). ChTX‐sensitive current (lower part in A) was obtained by mathematic subtraction of transmembrane current in the presence of ChTX 0.5 μM from transmembrane current in the Control. B: mean current densities (pA.pF‐1) of WT COS‐7 cells (upper part, n = 11) and COS‐7 cells stably expressing Kv1.2 channels (lower part, n = 11) were recorded in conventional whole‐cell configuration voltage step protocol (see above A); ● represents control, ▴ in the presence of ChTX 0.5 μM, and ○ ChTX‐sensitive current (Control ChTX 0.5 μM). Kv1.2‐C cells were treated with either EFs (100 mV/mm) only or EFs plus ChTX (0.5 μM) and the migration directedness (C) and speed (D) were analyzed, respectively. E,F: composing migration trajectories of Kv1.2‐C cells with EF (100 mV/mm) and EF + ChTX (0.5 μM) by pointing all the trajectories starting from the origin point on axes (0,0), respectively. Results are presented as means ± SE; n ≥ 3. *P < 0.05.

In order to pharmacologically identify the role Kv1.2 channels in the regulation of electrotaxis, we employed charybdotoxin (ChTX) which significantly suppressed Kv1.2 current in Kv1.2‐C cells (*P* < 0.05 in paired *t*‐test, see Fig. 4A,B). ChTX‐sensitive current obtained by arithmetic subtraction of the current in the presence of ChTX from the Control current was well fitted by Boltzmann function (V_0.5_ = 33.2 ± 2.3 mV, k = 24.6 ± 1.5), whereas ChTX‐sensitive current of WT COS‐7 cells was negligibly small (Fig. 4B, upper part). ChTX treatment significantly reduced cathodal migration directedness of Kv1.2‐C cells by twofold with the θ‐value reduction from 0.7 to 0.35. At the same time, ChTX did not affect the migration speed of the cells, suggesting that the motility of the cells was not compromised (Fig. 4C,D). These data indicate that inhibition of Kv1.2 channels reduces COS‐7 cell sensitivity of migration direction rather than motility during EF stimulation. Representative cell trajectories illustrate that Kv1.2 inhibitor impeded Kv1.2‐C cells cathodal migration (Fig. 4E,F).

### Kv1.2 channel interacts with cortactin to mediate directional migration

**Figure 5:**
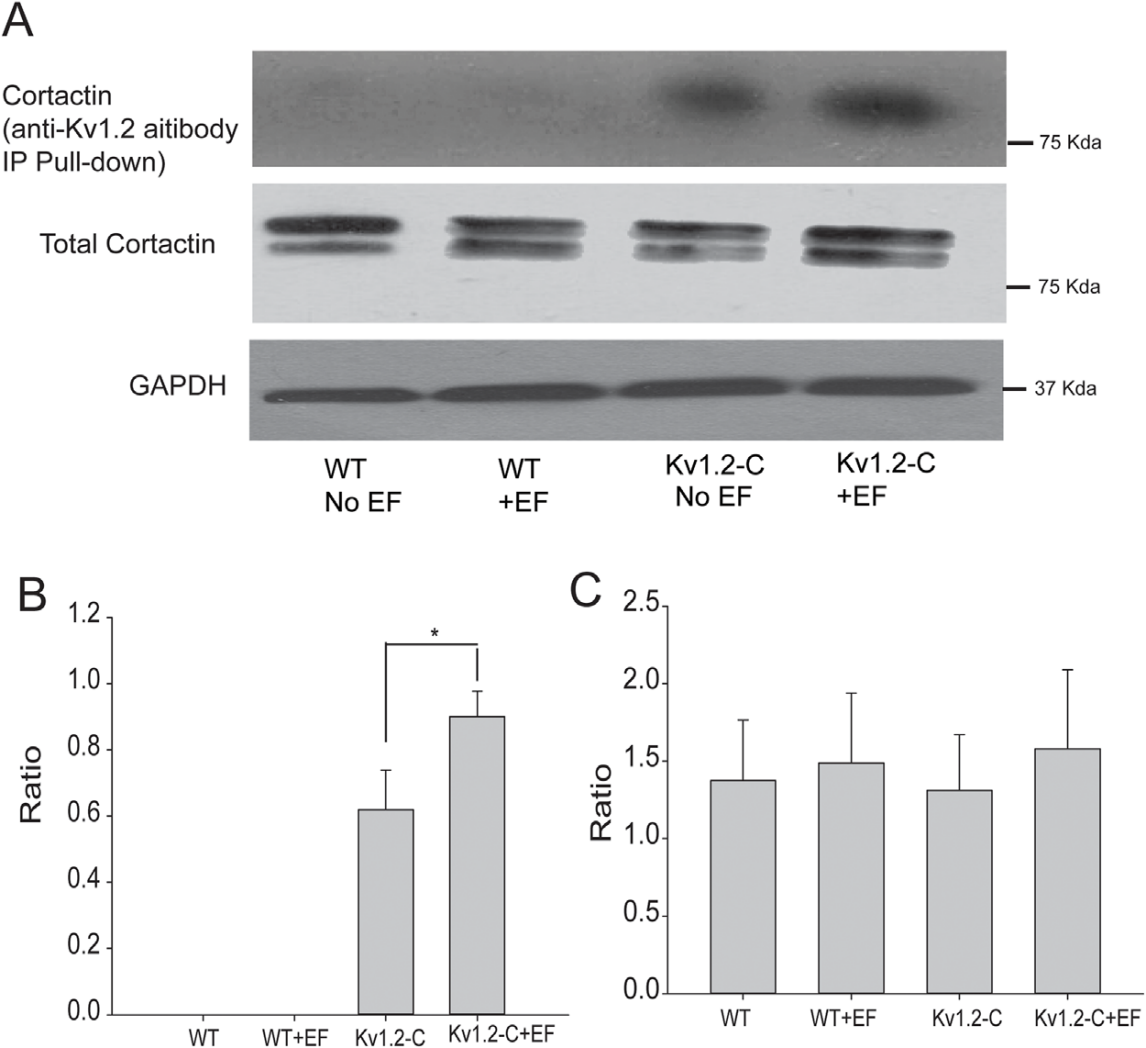
EF increased interaction between Kv1.2 channels and cortactin. The Protein A/G Argose‐Kv1.2 antibody immunoprecipitation (IP) pull‐down assay with indicated Kv1.2‐C cell lyses using cortactin antibody. The blot results show that EF increases the binding between Kv1.2 and cortactin. However, the interaction in WT cells was not observed even after EF stimulation (A and B). Additionally, neither overexpression of Kv1.2 nor EF treatment changed cortactin expression (A and C). Results are presented as means ± SE; n ≥ 3. *P < 0.05.

To explore the downstream effector responsible for the Kv1.2 signalling transduction during the regulation of the electrotactic response, we further investigated the potential involvement of the scaffolding protein cortactin in this event. WT COS‐7 and Kv1.2‐C COS‐7 cells were stimulated with 150 mV/mm EF, followed by cell lysis and pull‐down assay for the harvested protein. The anti‐Kv1.2 channel antibodies were used to pull‐down Kv1.2 channel together with any proteins with which it was binding. The result showed that EF triggered Kv1.2 channel binding to scaffolding protein cortactin (Fig. 5). These data also suggest that the Kv1.2 ‐cortactin interaction is greater in Kv1.2‐C COS‐7 cells than in WT COS‐7 cells, possibly due to the fact that Kv1.2‐C COS‐7 cells essentially expressed more Kv1.2 channel proteins which subsequently bound higher quantity of cortactin.

### Kv1.2 co‐localization with cortactin at the cathode‐facing membrane at electrotaxing cells

**Figure 6:**
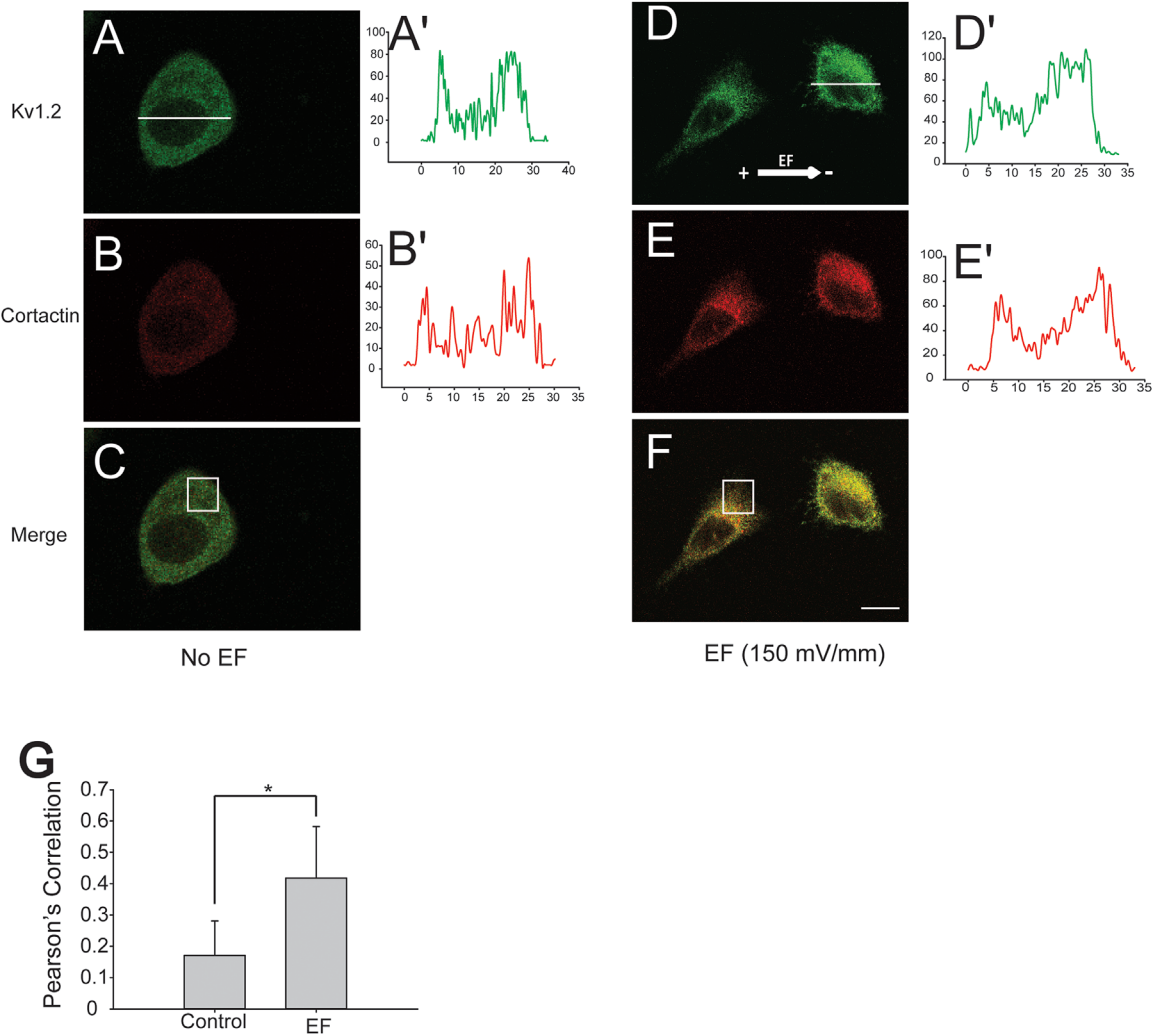
EF polarized Kv1.2 and cortactin co‐localization at the cathode‐facing membrane of migration cells. A–F: Confocal immunocytochemistry staining of Kv1.2 (green) and cortactin (red) on Kv1.2‐C cells in the absence or presence of EF (150 mV/mm) treatment, respectively. A',B'; D',E': the fluorescent profile of Kv1.2 and cortactin across the cell paralleling to EF vector were analysed accordingly with ImageJ software. White line indicates the analysed area of the fluorescence profile; A',B' show the fluorescence intensity profiles of the cells without EF, and D',E' with 150 mV/mm EFs for 2 h. G: co‐localization of Kv1.2 and cortactin at the cathode‐facing membrane on Kv1.2‐C clone in the absence or presence of EF stimulation. The white rectangles on C and F are the analyzed areas. Means ± SE; n ≥ 3. *P < 0.05.

It was well established that cortactin played a critical role in cell migration through its interaction with small GTPases and F‐actin (Kowalski et al., 2005; Van Rossum et al., 2006; MacGrath and Koleske, 2012), and that cortactin directly binds to Kv1.2 and modulates channel functions (Williams et al., 2007). The immunocytochemistry experiments were performed on EF‐stimulated COS‐7 cells to examine its effect on Kv1.2 localisation, and its co‐localization with cortactin during electrotaxis. In the control group both Kv1.2 and cortactin were evenly distributed around the cell membrane (Fig. 6A,B), whereas in EF‐stimulated group Kv1.2 and cortactin were polarized at the cathode‐facing membrane of the electrotaxing cells (Fig. 6C,D). In contrast with the control group (Fig. 6A',B'), the fluorescence intensity analysis also indicated that Kv1.2 and cortactin molecules were asymmetrically redistributed at the cathode side of the COS‐7 cells (Fig. 6C',D'). The defined areas on cell membrane were selected for co‐localization analysis using ImageJ (Fig. 6C,F). The results showed that EF significantly increased the co‐localization of Kv1.2 channels and cortactin at the cathode‐facing membrane of the electrotaxing cells in comparison to the control group (Fig. 6G).

## Discussion

In the present study we report that the voltage‐gated Kv1.2 channels play a key role in EF‐stimulated directional cell migration. Here we demonstrated that variation in the expression level of Kv1.2 channel had a pivotal effect on the directedness of the electrotactic response. In comparison to WT COS‐7 cells, Kv1.2‐C clone displayed the fastest elevation of the electrotactic response, as well as the highest migration directedness even when treated with EF as low as 50 mV/mm COS‐7. This is in agreement with the Kv1.2 expression pattern, with the Kv1.2‐C clone showing the high expression level compared to the control and other two clones, suggesting a Kv1.2 channel dependent electrotactic response.

Contribution of various types of ion channels to electrotaxis has been previously postulated (Djamgoz et al., 2001; Shanley et al., 2006; Huang et al., 2009), which suggested that ion channel‐dependent cell migration is a complex multicomponent mechanism. Voltage gated Ca^2+^‐channels were considered to play an important role in electrotaxis in slime mould (*Dictyostelium discoideum)*, which migrates cathodally in EF when Ca^2+^ flows into the cell, and inhibition of Ca^2+^ influx prevents electrotaxis (Shanley et al., 2006). However, mouse fibroblast cells displayed Ca^2+^ ‐independent type of electrotaxis (Brown and Loew, 1994), zebra fish keratinocytes showed that the Ca^2+^ spikes did not contribute to EF‐sensing machinery (Huang et al., 2009), and other studies in mammalian cells indicated that knocking out of a number of voltage‐gated Ca^2+^ channels did not affect electrotaxis (Brown and Loew, 1994). These findings suggested that Ca^2+^‐channels do not necessarily represent universal mechanism for electrotaxis in all mammalian cells (including COS‐7 cells) for several reasons: Firstly, the wild type COS‐7 cells should have responded to EFs when Ca^2+^ influxes into the cells. Secondly, if membrane depolarization induced by EFs activated Ca^2+^ channels the ChTX would not effectively block the response of Kv1.2‐C clone to EFs. And thirdly, we did not detect meaningful voltage‐gated Ca^2+^ channel activity in wild COS‐7 cells by direct patch‐clamp readouts.

Voltage‐gated Na^+^ channels have also been previously proposed to contribute towards the EF‐driven metastasis of rat prostate cancer cells (Djamgoz et al., 2001). Na^+^/H^+^ exchanger (NHE1) was suggested to transduce electric signals to phosphotydilinosotol‐3‐kinase (PI3K) and mediate EF‐dependent directional migration of epithelial cells (Zhao et al., 2006). However, since we did not observe any significant activity of Na^+^ channel in wild type COS‐7 cells, its effects on electrotaxis, therefore, may not be universal as well.

RhoA kinase is known as an important regulator of actin microfilament dynamics which is essential in the regulation of the mobility and contraction during cell migration (Cachero et al., 1998; Pertz et al., 2006). Physical interaction between RhoA and Kv1.2 channel indicates involvement of Kv1.2 channel in cell motility (Cachero et al., 1998). It was reported that guanine nucleotide‐bound RhoA could inhibit the activity of Kv1.2 channels, subsequently suppressing the cell movement (Williams et al., 2007). Furthermore, phosphorylation of C terminus of Kv1.2 channel by tyrosine residues, as well as mutation of these residues, inhibits Kv1.2 interaction with cortactin and activity of Kv1.2 channels (Hattan et al., 2002). On the other hand, cortactin regulates lamellipodia persistence through binding of F‐actin, Arp2/3, and cadherin at the leading edge (Kowalski et al., 2005).

In the Control cells with no EF stimulation, the leading edge of the lamellipodia/filopodia was generated spontaneously at random positions of the cell membrane without recordable spatial and temporal control, which subsequently established the chaotic or totally random migration. When the cells were exposed to the EF, the anodal facing cell membrane tends to depolarize as the negative charges are accumulated on the surface of the membrane (Djamgoz et al., 2001). Consequently, the positive charges would accumulate on the cathodal facing membrane which results in membrane hyperpolarization due to activity of K^+^ channels. Activation of K^+^ channels, for example, Kv1.3 channel has been reported to induce integrin‐mediated cell adhesion and migration (Kolaczkowska and Kubes, 2013). Our study suggests that over expression of Kv1.2 channel in clone C acts as an amplifier for EF sensing mechanism and confers greater electrical sensitivity for Kv1.2‐C COS‐7 cells. We propose that Kv1.2 channel is a key sensor regulating the leading edge formation at the cathodal facing membrane of the electrotaxing cells. Both Kv1.2 channel and cortactin are polarized cathodally and co‐localized at the leading edge cell membrane facing cathode, increasing the probability of interaction in the focal area, which is proved with the immunocytochemistry and pull down analysis. Interestingly, there is only one band appeared in pull down experiment compared to a doublet in whole cell lyses which is in agreement with other's report (Williams et al., 2007). The exact reason is still not clear, but one possibility is that Kv1.2 only binds to one band strong enough that can be assessed by the pull down assay. Cortactin has previously been described to be able to influence Kv1.2 channel activity at the leading edge where cortactin links up Kv1.2 channels with cytoskeleton proteins (Williams et al., 2007). In addition, cortactin targeting of Kv1.2 channels at the cell membrane is required for the Kv1.2 channel activation and cell hyperpolarization (Williams et al., 2007).

**Figure 7:**
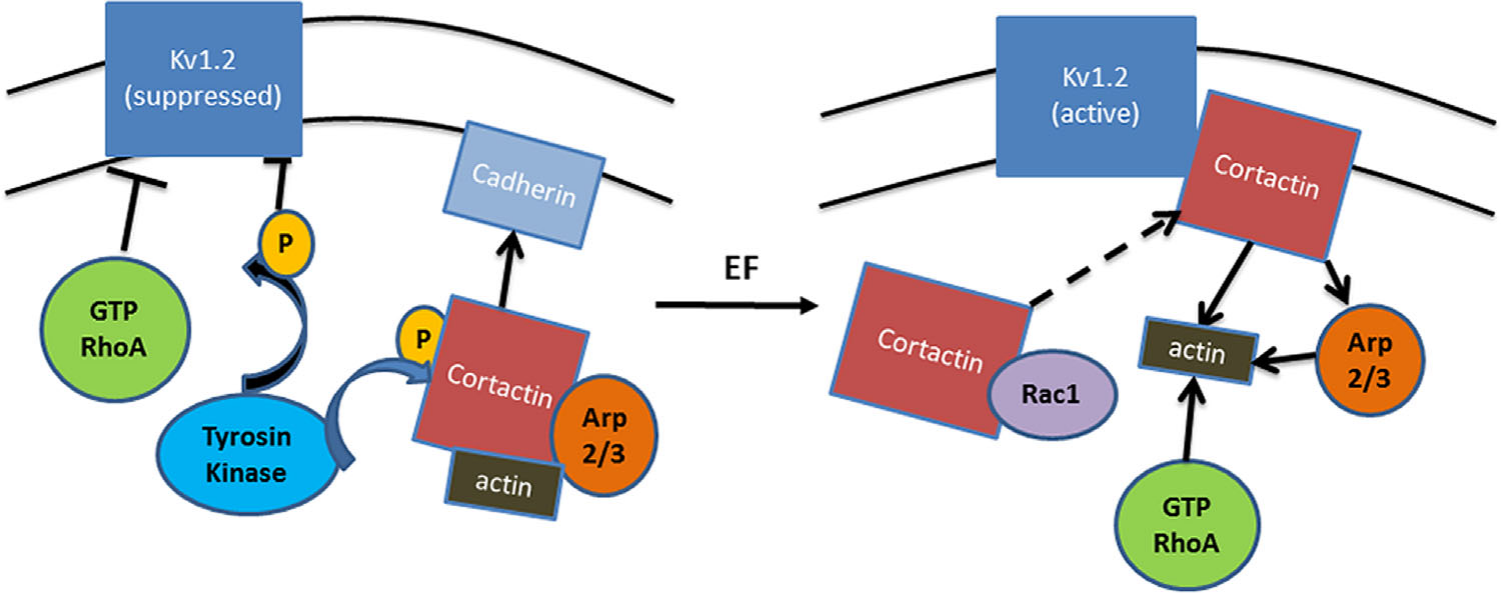
Schematic illustration of a hypothetical mechanism for Kv1.2 in electrotaxis. The most prominent function of Kv1.2 channel is to control the electrogenic transport of ions across the plasma membrane. It hyperpolarizes the cell membrane potential by elevating the extracellular K^+^ concentration. Cortactin is highly enriched in lamellipodia and enhances protrusion persistence, which potentially binds to F‐actin to regulate membrane dynamics and form adhesion nucleus through regulation of Arp2/3 and interaction with cadherin. The Kv1.2 can be suppressed by activated RhoA and the tyrosine phosphorylation at its C‐terminal which blocks its interaction to cortactin. When treated with EF, Kv1.2 channel may act as sensors responding to the electric signals, and subsequently activated Kv1.2 channel binds cortactin at the leading edge. The asymmetrical colocalization of Kv1.2 channel and cortactin at the cathode‐facing membrane of the migration cells enhances actin polymerization and protrusion thereby the directional migration is initiated.

EF‐dependent conformational changes of Kv1.2 channel may be essential for cortactin to transduce electric signal to the cytoskeleton. It is possible that EF stimulates activity of Kv1.2 channels and consequently reduces RhoA interaction with C terminus of Kv1.2 channel, which gives way for further cortactin binding at the leading edge of the electrotaxing cells. Cortactin also might activate Arp2/3 and form nucleation points for actin polymerization (Uruno et al., 2001). Kv1.2 channel, presumably, stays active for the entire period when EF stimulation is applied, thereby maintaining the biased protrusion and directional migration of the cells. Summarized mechanism of Kv1.2 channel mediated electrotaxis is shown in Figure 7.

Our data provide evidence that in the present model ChTX was an effective and selective antagonist of Kv1.2 current which was illustrated in both electrophysiological and cell migration (directness) experiments with no other conductance affected (Fig. 4B). ChTX‐sensitive component in transmembrane outward current of Kv1.2‐C clone was rather small: ∼20 % of the entire current amplitude, however, even small change in current may result in significant change in potential which was consistently proved in Figure 2D. ChTX caused no effects on current amplitude in the Wild Type showing no trace of ChTX‐sensitive component in these Wild Type COS‐7 cells in the absence of forced expression of Kv1.2 channels (Fig. 4B). According to the Western blots (Fig. 1A) Wild Type COS‐7 cells had certain level of expression of Kv1.2 channels, however, their functional appearance was negligible, which may be due to rather low conductance of Kv1.2 channels 14–18 pS (Hart et al., 1993; Conforti et al., 2000). In the same time it comes no surprise that the Wild Type COS‐7 cells displays no significant Kv1.2 current since even high level of Kv1.2 expression in Kv1.2‐C clone yielded with an outward current augmented only by 20%.

In this study, we propose a model of EF‐dependent re‐localization of Kv1.2 channels at the cathode‐facing membrane of electrotaxing cells, where cortactin coordinates protein binding of Kv1.2 channel with actin in cytoskeleton and establishes a directional migration. Over expression of Kv1.2 channels or pharmacological inhibition respectively resulted in increased and reduced electrotactic response, respectively, indicating pivotal role of Kv1.2 channels in EF‐sensing. By identification of molecular mediators involved in electrotaxis, this study also provides potential applications in the regulation of cancer cell metastasis, and the guidance of the targeted cell migration in cell replacement therapy and wound healing.
